# Small Interfering RNA Inhibition of Andes Virus Replication

**DOI:** 10.1371/journal.pone.0099764

**Published:** 2014-06-12

**Authors:** Cheng-Feng Chiang, Cesar G. Albariňo, Michael K. Lo, Christina F. Spiropoulou

**Affiliations:** Viral Special Pathogens Branch, Division of High-Consequence Pathogens and Pathology, Centers for Disease Control and Prevention, Atlanta, Georgia, United States of America; University of Georgia, United States of America

## Abstract

Andes virus (ANDV) is the most common causative agent of hantavirus pulmonary syndrome (HPS) in the Americas, and is the only hantavirus associated with human-to-human transmission. Case fatality rates of ANDV-induced HPS are approximately 40%. There are currently no effective vaccines or antivirals against ANDV. Since HPS severity correlates with viral load, we tested small interfering RNA (siRNA) directed against ANDV genes as a potential antiviral strategy. We designed pools of 4 siRNAs targeting each of the ANDV genome segments (S, M, and L), and tested their efficacy in reducing viral replication *in vitro*. The siRNA pool targeting the S segment reduced viral transcription and replication in Vero-E6 cells more efficiently than those targeting the M and L segments. In contrast, siRNAs targeting the S, M, or L segment were similar in their ability to reduce viral replication in human lung microvascular endothelial cells. Importantly, these siRNAs inhibit ANDV replication even if given after infection. Taken together, our findings indicate that siRNAs targeting the ANDV genome efficiently inhibit ANDV replication, and show promise as a strategy for developing therapeutics against ANDV infection.

## Introduction

Andes virus (ANDV), a New World pathogenic hantavirus, causes hantavirus pulmonary syndrome (HPS) in humans, with case fatality rates of 40% [1], [2]. ANDV is the major cause of HPS in South America, and has been associated with the most HPS cases to date. It has been classified as a category A pathogen, and is the only hantavirus known to be capable of human-to-human transmission [3], [4]. ANDV, a member of the *Bunyaviridae* family, is an enveloped virus with a tri-segmented, negative-sense, single-stranded RNA genome of approximately 11 kb [5]. The small (S), medium (M), and large (L) segments encode the nucleocapsid (N) protein, 2 glycoproteins (Gn and Gc), and an RNA-dependent RNA polymerase (RdRp or L-protein), respectively. N interacts with host mRNA and viral RNA during viral replication. Gn and Gc oligomerize to form spikes on the virus particle, mediating receptor binding and fusion with target cells. The L protein is responsible for replicating and transcribing the viral genome.

ANDV infection in humans occurs by exposure to excreta from the persistently-infected rodent reservoir [5]. The disease is characterized initially by fever, muscle aches, and headaches, followed by pulmonary edema due to vascular leakage. Patients with severe disease quickly develop respiratory failure or shock, often leading to death [6]. Levels of ANDV RNA peak at the time of pulmonary edema [7], [8], and viremia levels correlate with HPS severity [9]. Currently, no vaccines or antiviral drugs are approved to prevent or to treat HPS. Attempts to treat HPS with intravenous ribavirin have been ineffective after hospitalization [10], indicating that the final clinical stages of HPS progress too rapidly for ribavirin to exert an antiviral effect. However, no firm conclusions can be drawn from these studies given the low number of patients enrolled.

RNA interference (RNAi) is a post-transcriptional, sequence-specific RNA degradation process observed in eukaryotic cells, and is considered a defense mechanism against viral infection [11], [12]. Upon recognizing exogenous double-stranded RNA, the cytosolic ribonuclease Dicer cleaves it into small interfering RNAs (siRNAs) 21–25 nt in length. These siRNAs are incorporated into the RNA-induced silencing complex (RISC), in which siRNAs directly bind to complementary mRNA sequences to induce their cleavage, consequently silencing the expression of the targeted gene [13].

The major advantage of siRNA treatment is its target specificity. It has been shown that RNAi targeting viral genes inhibits viral replication *in vitro* and has been explored as a strategy to combat viral infection caused by, *e.g*., HIV-1, poliovirus, nairovirus, and Lassa virus [14]–[17]. RNAi-based therapy effectively reduces viral loads and increases survival rates in humans and animals infected with a number of other viruses [18]–[21]. Here, we investigate the potential of using siRNA against ANDV infection. Our data suggest that siRNAs targeting the ANDV genome can efficiently lower virus titers, thus showing promise as potential *in vivo* therapeutic agents against HPS.

## Materials and Methods

### Cell lines and viruses

African green monkey kidney (Vero-E6) cells were obtained from ATCC and maintained in DMEM (Life Technologies, Grand Island, NY, USA) supplemented with 10% heat-inactivated fetal bovine serum (FBS; Hyclone, Logan, UT, USA). Human lung microvascular endothelial cells (HMVEC-L; Lonza/Clonetics, Walkersville, MD, USA) were grown with EGM-2MV medium (Lonza/Clonetics) in cell culture flasks pre-coated with phosphate-buffered saline (PBS) containing 0.2% gelatin (Sigma-Aldrich, St. Louis, MO, USA). ANDV (strain Chile 9717869) was propagated in Vero-E6 cells in a biosafety level 3 laboratory. Viral titers were determined using immunostaining as described in the Immunofocus assays section.

### Transfection of plasmid and siRNA

Vero-E6 cells were transfected with a plasmid containing ANDV-GPC [22] using *Trans*IT-LT1 (Mirus Bio, Madison, WI, USA). siRNA sequences targeting ANDV S, M, and L segments were designed by Dharmacon (Lafayette, CO, USA) based on NCBI reference sequence database entries (assession numbers NC_003466.1, NC_003467.2, and NC_003468.2, respectively). The siRNA sequences are listed in Table S1. Vero-E6 cells and HMVEC-L were transfected with 100 nM siRNA using 0.2% and 0.05% DharmaFECT-1 (Dharmacon), respectively. Incubation times are indicated in figure legends.

### Western blotting

Vero-E6 cells or HMVEC-L were seeded at a density of 2×10^5^ cells/well in 12-well plates, or 5×10^5^ cell/well in 6-well plates, and incubated for 24 h. After transfection and ANDV infection, cells were lysed in RIPA buffer (Sigma-Aldrich). Lysate samples containing an equivalent of 5 µg protein were loaded and separated on NuPAGE 4–12% Bis-Tris gels (Life Technologies), then transferred onto PVDF membranes. After blocking with PBS containing 0.05% v/v Tween-20 and 5% w/v skim milk, the membranes were incubated overnight at 4°C with mouse monoclonal antibodies against Puumala virus N protein (1∶4000), ANDV Gc protein (1∶2000; US Biological, Swampscott, MA, USA), or β-actin (1∶4000; Sigma-Aldrich). Samples were washed in PBS containing 0.05% Tween-20 before adding horseradish peroxidase (HRP)-conjugated secondary antibody, and developed using SuperSignal West Dura Extended Duration Substrate (Thermo Scientific, Pittsburgh, PA, USA). Image analysis and densitometry were performed on the public domain program NIH ImageJ.

### Immunolabeling and immunofluorescence assays

The immunolabeling method used in this study was adapted from [23], with minor modifications. Vero-E6 cells or HMVEC-L were seeded at a density of 1×10^4^ or 2×10^4^ cells/well in 96-well plates, respectively, and incubated for 24 h. After siRNA transfection and ANDV infection, plates were washed 3 times with PBS containing 0.1% v/v Tween-20 (PBS-T), blocked with 5% skim milk in PBS-T, and incubated at 37°C for 30 min. Plates were then washed twice with PBS-T, incubated with anti-N protein antibody (mouse monoclonal anti-Puumala virus N protein, 1∶4000) for 30 min at 37°C, and washed as above. Plates were incubated with 1% H_2_O_2_ (Sigma-Aldrich) for 15 min at room temperature, and washed as above. For immunolabeling, goat anti-mouse IgG1-conjugated HRP (1∶25,000; SouthernBiotech, Birmingham, AL, USA) was added for 30 min at 37°C before washing as above. Signal was detected with chemiluminescent peroxidase substrate-3 (CPS-3, Sigma-Aldrich) on a Synergy HT Microplate Reader (Biotek, Broadview, IL, USA) using 0.1 s integration. For immunofluorescence, goat anti-mouse IgG1-FITC (1∶1000, SouthernBiotech) was used as the secondary antibody. Images were obtained with a fluorescence microscope (Nikon, Melville, NY, USA) using 10× magnification; all pictures were captured using the same exposure and gain settings.

### Cell viability assays

Cytotoxic effects of the siRNA pools in Vero-E6 and HMVEC cells were determined using the CellTiter-Glo Luminescent assay (Promega, Madison, WI, USA) according to the manufacturer's instructions. Briefly, after cells were treated with siRNA and infected with ANDV, CellTiter-Glo reagent was added to each well of a 96-well plate. After 10 min incubation at room temperature, luminescent signals were read as above.

### Immunofocus assays

The virus titers were determined by an immunostaining plaque assay. Viral plaques were detected using anti-Puumala virus N protein and rabbit anti-mouse IgG (H+L) conjugated with Alex Fluor 488 antibodies (Life Technologies), and counted under a fluorescence microscope.

## Results

### siRNA inhibits ANDV protein synthesis

**Figure 1:**
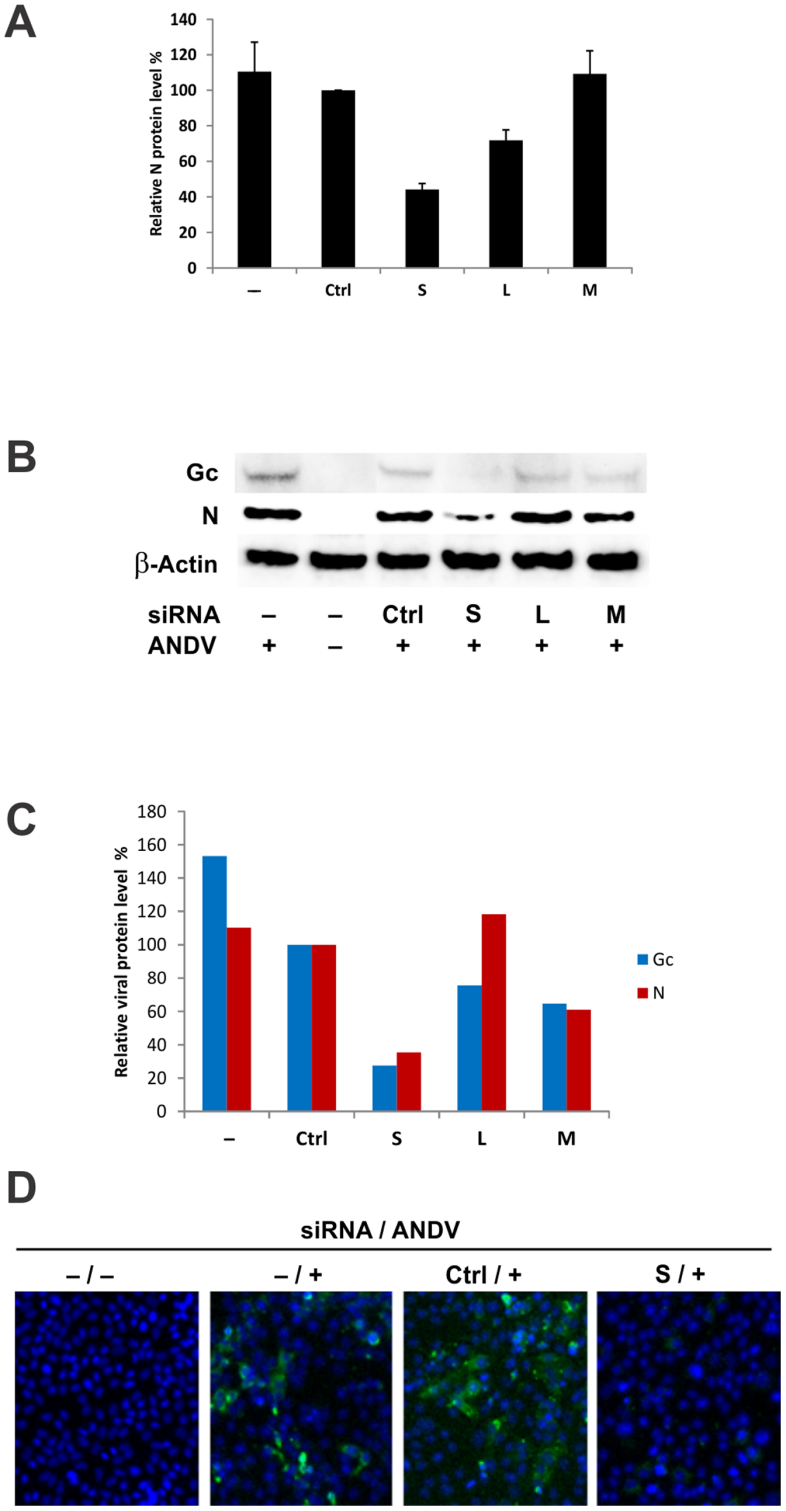
siRNA inhibits ANDV protein synthesis. Vero-E6 cells were transfected with 100 nM of either scrambled siRNA (Ctrl) or pools of siRNAs targeting the small (S), large (L), or medium (M) segment of ANDV using DharmaFECT 1. After 6 h, siRNAs were removed, and cells were infected with ANDV at multiplicity of infection (MOI)  = 0.5 for 48 h. (A) Cells were lysed, and expression of the N protein was quantitated by an immunolabeling assay. (B) The levels of Gc glycoprotein, N protein, and β-actin were analyzed by Western blotting, and quantified by densitometry. (C) Data are presented as % of scrambled siRNA control, and are normalized to β-actin internal controls. (D) N protein was detected by immunofluorescence staining 48 h post-infection. Green: N protein; blue: DAPI.

To determine if siRNA against the ANDV genome inhibits ANDV infection *in vitro*, we generated siRNA pools targeting each of the 3 ANDV genomic segments (Table S1). Each siRNA pool included 4 individual siRNAs targeting 4 separate regions of each virus segment. Vero-E6 cells were transfected with each siRNA pool prior to ANDV infection. We found that siRNA targeted against the S segment (siS) greatly reduced levels of viral protein expression. siS reduced ANDV N level by >60% (relative to non-targeting scrambled siRNA controls) as measured by immunolabeling (Figure 1A), and reduced N and Gc protein levels by >70% and 40%, respectively, as measured by Western blot analysis (Figure 1B and 1C). Furthermore, siS-induced reduction of N protein expression can be readily observed directly by immunofluorescence staining (Figure 1D).

We also found that siL marginally reduced levels of ANDV N and Gc by 28% and 25%, respectively (Figure 1A and C). While the siM pool reduced Gc protein levels by 36%, it had no effect on N protein levels (Figure 1A, C). The weak inhibition by the siM pool was not due to poor design of the siRNAs, as these same siRNAs completely suppressed virus glycoprotein synthesis when Gc was expressed from an ANDV GPC expression plasmid (Figure S1).

Cell viability assays showed no significant cytotoxicity after transfection with any of the siRNA pools, nor after 48 h of ANDV infection (data not shown). These initial results indicate that siRNAs targeting the ANDV genome can block ANDV infection, with the S segment being the most efficient target for inhibition in Vero-E6 cells.

### siRNA inhibits production of infectious ANDV

**Figure 2:**
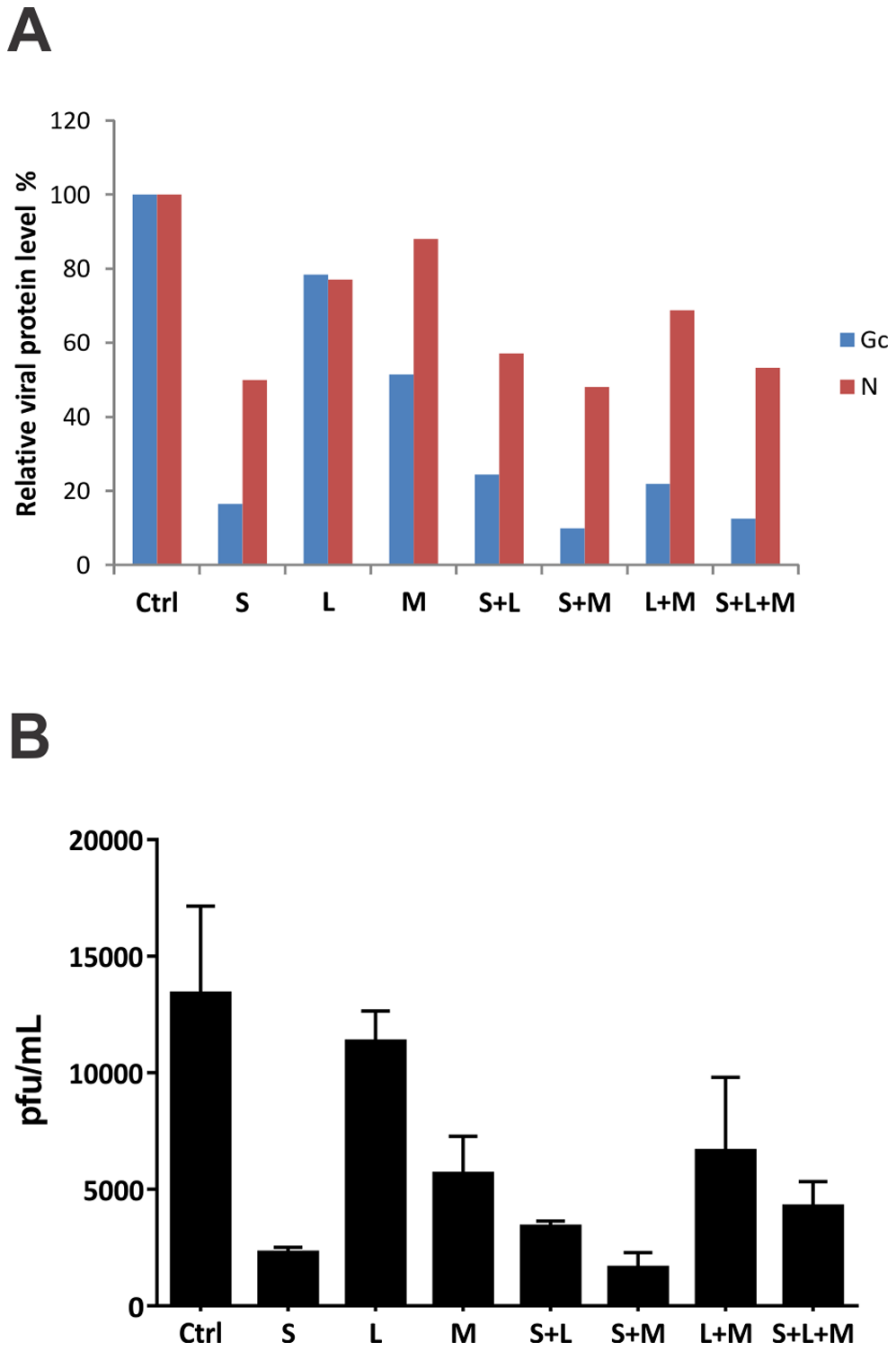
siRNA inhibits production of infectious ANDV. (A) Viral protein levels determined by Western blot analysis. Vero-E6 cells were transfected with 33 nM of each siRNA pool (S, L, or M) alone or in combination (S+L, S+M, L+M, or S+L+M) for 6 h. The total concentration of each transfection was brought up to 100 nM with scrambled siRNA. Cells treated with only scrambled siRNA were used as control. The cells were then infected with ANDV at MOI  = 0.5. ANDV Gc and N protein levels were determined by densitometry. (B) ANDV production and release were determined by immunofocus assays. All experiments were performed in quadruplicate.

To test whether these siRNA pools could act synergistically, we also transfected Vero-E6 cells with combinations of the siRNA pools (Figure 2A). Despite using less of each siRNA (33 nM), the individual siRNA pools gave similar results (Figure 2A) to those seen in the initial experiments using 100 nM of siRNA (Figure 1). siS inhibited N and Gc protein expression by over 50% and 80%, respectively, whereas siL and siM were less potent.

In addition, while treatment with either siS or siM pools individually inhibited infectious virus production by over 83% or 50%, respectively (Figure 2B), combinations of the 3 siRNA pools did not significantly enhance the inhibitory effect. We conclude that in Vero-E6 cells, pretreatment with the siS pool was the most effective for blocking ANDV protein synthesis and infectious virus release.

### siRNA inhibits ANDV replication and release when administered post-infection

**Figure 3:**
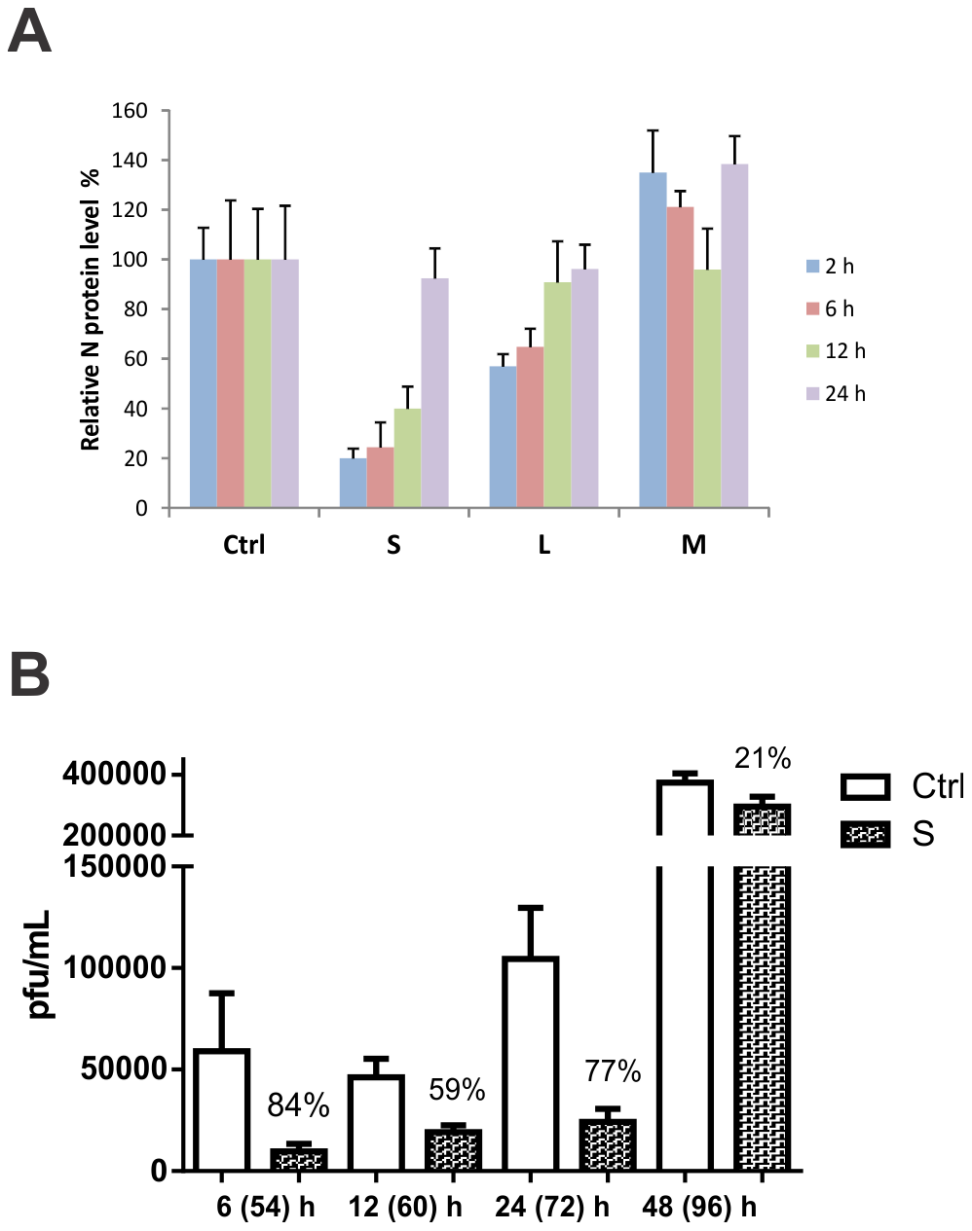
siRNA inhibits ANDV replication and release when administered post-infection in Vero-E6 cells. Vero-E6 cells were infected with ANDV at MOI  = 0.5. After virus adsorption for 2 h, the virus inoculum was removed and replaced with fresh media. Cells were then transfected with 100 nM siRNA 2, 6, 12, or 24 h post-infection. Time shown in parentheses indicates the total h post-infection. (A) N protein levels as determined by immunolabeling assays. (B) Infectious virus release as measured by immunofocus assays. After virus adsorption for 2 h, virus was removed and fresh media replaced. The cells were transfected with 100 nM siS for 6, 12, 24, or 48 h post-infection. Cell supernatants were harvested after 2 days, and infectious virus production was determined by immunofocus assays. All experiments were performed in triplicate. Data indicated above each bar represent percentage reduction in comparison to scrambled siRNA control.

To explore the potential for using siRNAs to treat already initiated ANDV infections, we sought to determine how long after ANDV infection we can transfect each siRNA pool and still reduce virus replication. Transfecting with siS 2, 6, or 12 h after ANDV infection resulted in a dramatic decrease of virus protein synthesis, as demonstrated by N protein reduction by over 70, 60, or 40%, respectively (Figure 3A). Transfecting siL 2 or 6 h post-infection modestly decreased N protein expression by 30%, while the siRNA pool targeting M had no effect on virus protein synthesis (Figure 3A).

Since siS was again the most effective siRNA pool, we tested its effect on virus titers. siS transfected 24 h post-infection had the strongest inhibitory effect, reducing viral production by over 75% (Figure 3B). The efficiency of siS was lower when transfected 48 h post-infection, with 21% inhibition (Figure 3B). These results indicate that siRNA pools targeting the S segment can effectively block ANDV replication and virus release *in vitro* even if administered 24 h post infection.

### siRNA inhibits ANDV replication in human primary lung endothelial cells

**Figure 4:**
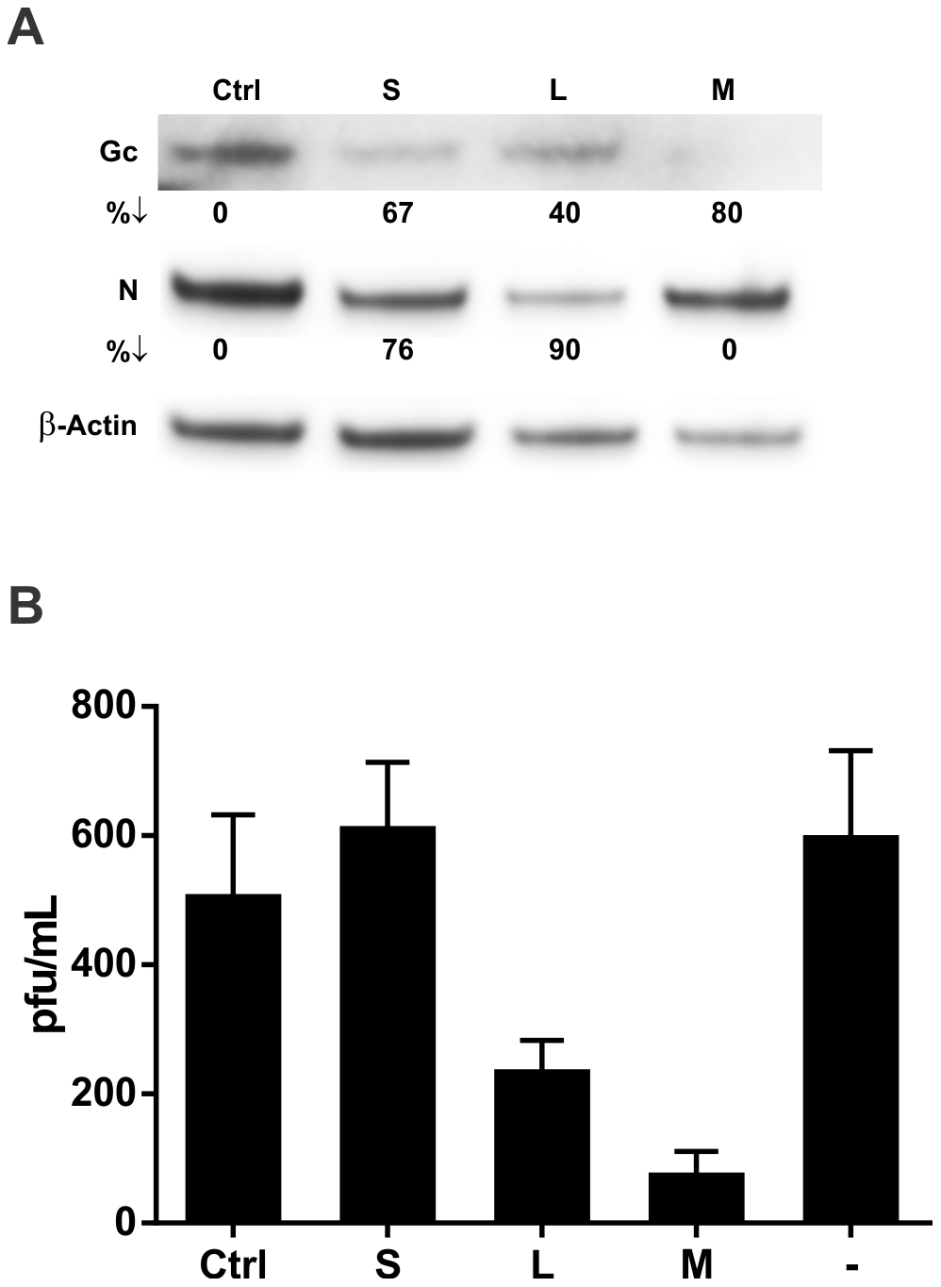
siRNA inhibits ANDV replication in human primary lung endothelial cells (HMVEC-L). HMVEC-L were transfected with siRNAs for 6 h, and then infected with ANDV at MOI  = 0.5. After 2 h of virus adsorption, the virus inoculum was removed and replaced with fresh media. (A) Viral protein levels as determined by Western blotting. Percent downregulation (%↓) of N or Gc protein represents percent decrease compared to non-targeting siRNA control. (B) Viral production as measured by immunofocus assays 48 h post-infection.

As lung microvascular endothelial cells are the primary cellular targets of ANDV infection *in vivo*, we were interested in testing whether similar siRNA inhibitory effects could be obtained in HMVEC-L. As in the initial Vero-E6 cell experiments, we first examined the effects of treating HMVEC-L with the 3 siRNA pools prior to ANDV infection. Inhibitory effects were observed, but the relative efficiency of the siRNA pools differed from the effects seen in the Vero-E6 continuous cell line. In HMVEC-L, siM inhibited Gc expression by 80% (Figure 4A), and reduced infectious virus titers by >85%. In addition, siL reduced both Gc and N protein levels (40 and 90% reduction, respectively; Figure 4A), and reduced infectious virus titers by 50%. Surprisingly, while siS reduced Gc and N protein levels (67 and 76%, respectively; Figure 4A), it failed to inhibit viral release from HMVEC-L (Figure 4B). This difference is likely related to the significantly different virus replication dynamics and cellular localization of virus protein pools in lung endothelial cells versus in the Vero-E6 continuous cell line, as ANDV-infected HMVEC-L released >20-fold less virus than infected Vero-E6 cells (Figure 2B and 4B).

### siRNA administered to HMVEC-L post-infection inhibits ANDV replication and infectious virus release

**Figure 5:**
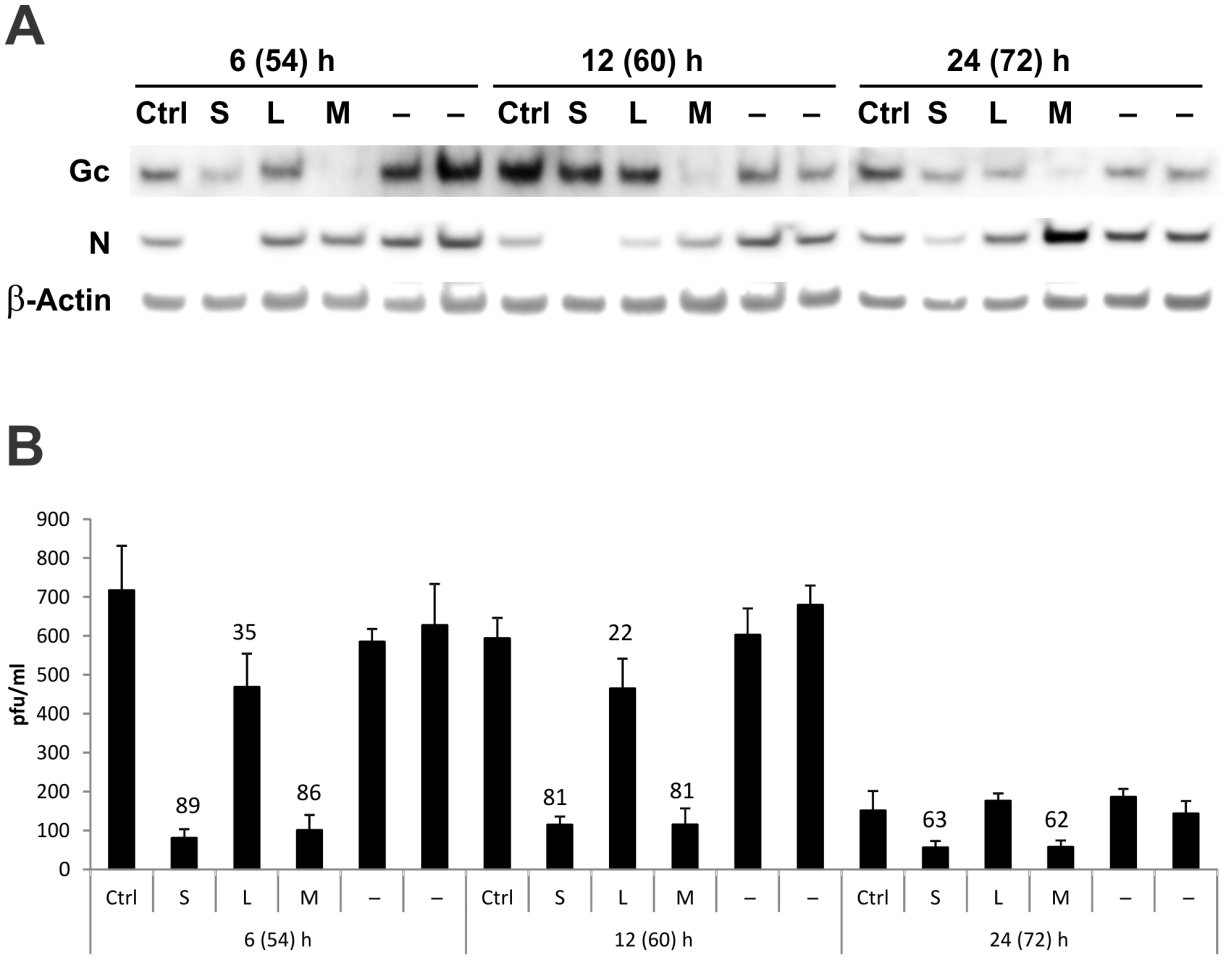
siRNA administered to HMVEC-L post-infection inhibits ANDV replication and infectious virus release. Cells were infected with ANDV at MOI  = 0.5. After virus adsorption for 2 h, the virus inoculum was removed and replaced with fresh media. The cells were then transfected 6, 12, or 24 h post-infection with 100 nM of siS, siL, or siM using DharmaFECT 1. Time shown in parentheses indicates total h post-infection. (A) Viral protein levels as determined by Western blotting. (B) Viral release as determined by immunofocus assays. All experiments were performed in triplicate. Data shown above each bar indicates percent decrease in comparison to the scrambled siRNA control.

Finally, we determined the effects of treating HMVEC-L with siRNA at various times after ANDV infection. We found that siS transfected 6, 12, and 24 h post-infection reduced virus N protein levels by more than 50% in comparison to the scrambled control siRNA treatment (Figure 5A). siM transfected 6, 12, and 24 h post-infection reduced virus Gc protein levels by more than 80%. More importantly, siS or siM alone significantly inhibited virus production in HMVEC-L when transfected 6–24 h post-infection (Figure 5B).

## Discussion

To determine whether siRNA has potential as a therapeutic agent against ANDV, we tested pools of siRNAs targeting the ANDV genome. These pools were tested *in vitro* in both continuous and primary cell lines. The siS pool targets the virus S segment, which encodes the virus N protein. Treatment with this siRNA pool very efficiently reduced virus protein levels, a result consistent with previous findings in other bunyaviruses [14], [24]–[26]. The N mRNA can be detected as early as 2 h post ANDV infection, and is the first viral RNA detected during infection [27], [28]. The N protein has several important roles in viral replication, as it encapsidates and protects viral RNA [29]–[31], and participates in initiating viral transcription and translation by binding cellular 5′ mRNA caps [32]. N protein gradient in the host cell cytoplasm also determines the switch from viral transcription to replication [33]. Based on all these critical functions of N in the virus life cycle, it is not surprising that siS knockdown of the S segment readily decreased virus replication.

Another protein important for virus replication is the L protein. L mRNA is the least abundant during infection, so we anticipated that it could be more efficiently suppressed by siRNA, leading to a significant decrease of ANDV replication. To our surprise, siL had minimal effects on viral protein synthesis and virus release in Vero-E6 cells. Similar to siL, siM only modestly reduced protein levels in Vero-E6 cells. This weak inhibition by siM was not the result of designing ineffective siRNAs, since siM completely suppressed Gc protein when Gc was expressed from an ANDV GPC plasmid (Figure S1). Surprisingly, co-transfection of siS and siM, or siS and siL, did not suppress viral protein expression and virus production any more than did siS alone. Overall, the results reported here for ANDV infection of a continuous cell line (Vero-E6) are similar to previous findings that siRNAs targeting the L and M segments of other bunyaviruses are weaker inhibitors than those targeting the S segment [14], [25], [34].

Vascular endothelial cells are the main target cells of ANDV infection in humans [35]. To our surprise, the pattern of viral replication inhibition observed using the 3 siRNAs pools in primary human lung cells was different from that observed in Vero-E6 cells. While siM minimally affected ANDV growth in Vero-E6 cells, it very efficiently inhibited virus protein expression (80%), and, more importantly, infectious virus release (86%) in HMVEC-L. This reduction of virus replication was not due to the induction of IFN-β by the siM (data not shown). The differing abilities of the siRNAs to inhibit ANDV replication in Vero-E6 cells compared with HMVEC-L are likely related to differences in virus replication dynamics and protein pools in these different cells. Unlike in Vero-E6 cells, ANDV titers in endothelial cells are relatively low despite considerable accumulation of intracellular viral proteins [36]. In addition, unlike in Vero-E6 cells, viral glycoproteins are detected mainly in the lysosome rather than at the cell surface in endothelial cells [36], [37]. It is plausible that in endothelial cells, viral glycoproteins are a limiting factor for virus production, and reducing the glycoprotein levels with siM has greater impact on virus replication and release. Such significantly different siRNA inhibitory profiles between a continuous cell line that supports ANDV growth and primary lung endothelial cells (a target relevant to natural human infections) stress the importance of testing siRNAs in a variety of infection settings.

While the delivery of siRNA is still a challenge to their actual clinical use, the ability of siRNAs to efficiently block ANDV replication up to 24 h post-infection is very encouraging. Although suggesting the use of siRNA as a treatment for HPS based on the *in vitro* results presented here is quite premature, our study can be considered as proof of principle that siRNAs directed against ANDV genome can effectively lower virus replication and infectious virus release. As ANDV viremia levels correlate with HPS severity, and ANDV RNA peaks at the time of pulmonary edema, siRNA suppression has potential as a therapeutic HPS treatment [7]–[9].

## Supporting Information

Figure S1 **Silencing efficiency of siM.** Vero-E6 cells were mock-transfected or transfected using TransIT-LT1 with 2 µg of pCAGGS-GPC (pM) for 24 h, and then transfected with 100 nM of non-targeting control, siM, or mock-transfected for 2 days. Cells were subsequently lysed, and Gc and β-actin levels determined by Western blotting.
(TIF)

Table S1 **siRNAs targeting Andes virus (ANDV) genome.** siRNAs were designed based on NCBI reference sequences NC_003466.1, NC_003467.2, and NC_003468.2 for ANDV S, M, and L segments, respectively.
(DOCX)
