# Supplementary figures and images for: Small Interfering RNA Inhibition of Andes Virus Replication
Source: PLoS One. 2014 Jun 12;9(6):e99764. doi: 10.1371/journal.pone.0099764 (PMC4055710; doi:10.1371/journal.pone.0099764)

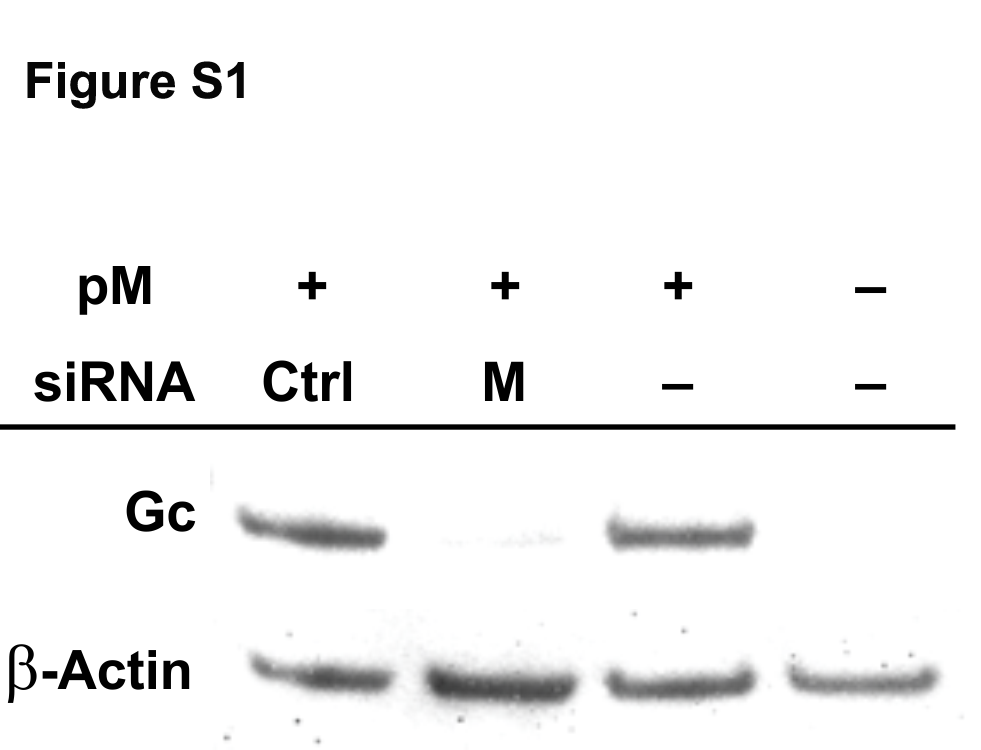

Supplement: Figure S1 — Silencing efficiency of siM. Vero-E6 cells were mock-transfected or transfected using TransIT-LT1 with 2 µg of pCAGGS-GPC (pM) for 24 h, and then transfected with 100 nM of non-targeting control, siM, or mock-transfected for 2 days. Cells were subsequently lysed, and Gc and β-actin levels determined by Western blotting. (TIF) [file pone.0099764.s001.tif]
