# Supplementary material for: Small Interfering RNA Inhibition of Andes Virus Replication
Source: PLoS One. 2014 Jun 12;9(6):e99764. doi: 10.1371/journal.pone.0099764 (PMC4055710; doi:10.1371/journal.pone.0099764)
Supplement: Table S1 — siRNAs targeting Andes virus (ANDV) genome. siRNAs were designed based on NCBI reference sequences NC_003466.1, NC_003467.2, and NC_003468.2 for ANDV S, M, and L segments, respectively. (DOCX) [file pone.0099764.s002.docx]

**Table S1**

| **siRNAs targeting Andes virus (ANDV) genome** | | | |
| --- | --- | --- | --- |
| Pool | siRNA | Sequence (5′ to 3′) | Targeted region in ORF |
| siS | S1 | Sense: GGU CAA AGC CCG AAA UAU AUU  Antisense: UAU AUU UCG GGC UUU GAC CUU | 669–687 |
|  | S2 | Sense: GGA AAU GGG AGG UGG GUU AUU  Antisense: UAA CCC ACC UCC CAU UUC CUU | 1528–1546 |
|  | S3 | Sense: GGA UAA GGU UUA AGG AUG AUU  Antisense: UCA UCC UUA AAC CUU AUC CUU | 509–527 |
|  | S4 | Sense: GUA AAG AAG CUG UGA AUC AUU  Antisense: UGA UUC ACA GCU UCU UUA CUU | 1205–1223 |
| siM | M1 | Sense: GAA CAA ACU UGC AAG CAU AUU  Antisense: UAU GCU UGC AAG UUU GUU CUU | 2494–2512 |
|  | M2 | Sense: GGG AAG GCC UAU AAG AUA AUU  Antisense: UUA UCU UAU AGG CCU UCC CUU | 2431–2449 |
|  | M3 | Sense: CAG UGA AAG UAG UUG GUA AUU  Antisense: UUA CCA ACU ACU UUC ACU GUU | 3143–3161 |
|  | M4 | Sense: AGU GAA AGU AGU UGG UAA AUU  Antisense: UUU ACC AAC UAC UUU CAC UUU | 3144–3162 |
| siL | L1 | Sense: GGU CAU GAG UAU UGA UUU AUU  Antisense: UAA AUC AAU ACU CAU GAC CUU | 1712–1730 |
|  | L2 | Sense: CAG AAG ACA UUG AGG UUA AUU  Antisense: UUA ACC UCA AUG UCU UCU GUU | 6094–6112 |
|  | L3 | Sense: AGG UAA AGU UCA AUG GAA AUU  Antisense: UUU CCA UUG AAC UUU ACC UUU | 4037–4055 |
|  | L4 | Sense: CAG CCA AUG UAA AGG GAA AUU  Antisense: UUU CCC UUU ACA UUG GCU GUU | 3184–3202 |
